# Peroxisome proliferator-activated receptor*β*/*δ* activation is essential for modulating p-Foxo1/Foxo1 status in functional insulin-positive cell differentiation

**DOI:** 10.1038/cddis.2015.88

**Published:** 2015-04-09

**Authors:** L Li, T Li, Y Zhang, Z Pan, B Wu, X Huang, Y Zhang, Y Mei, L Ge, G Shen, R-s Ge, D Zhu, Y Lou

**Affiliations:** 1 Institute of Pharmacology, Toxicology and Biochemical Pharmaceutics, Key Innovation Team for Stem Cell Translational Medicine of Cardiovascular Disease of Zhejiang Province, College of Pharmaceutical Sciences, Zhejiang University, Hangzhou, China; 2 Cardiovascular Key Laboratory of Zhejiang Province, The 2nd Affiliated Hospital, College of Medicine, Zhejiang University, Hangzhou, China; 3 The Population Council at the Rockefeller University, New York, NY 10021, USA; 4 Institute of Reproductive Biomedicine, the 2nd Affiliated Hospital, Wenzhou Medical University, Wenzhou, China

## Abstract

Peroxisome proliferator-activated receptors (PPARs) participate in energy homeostasis and play essential roles in diabetes therapy through their effects on non-pancreas tissues. Pathological microenvironment may influence the metabolic requirements for the maintenance of stem cell differentiation. Accordingly, understanding the mechanisms of PPARs on pancreatic *β*-cell differentiation may be helpful to find the underlying targets of disrupted energy homeostasis under the pancreatic disease condition. PPARs are involved in stem cell differentiation via mitochondrial oxidative phosphorylation, but the subtype member activation and the downstream regulation in functional insulin-positive (INS^+^) cell differentiation remain unclear. Here, we show a novel role of PPAR*β/δ* activation in determining INS^+^ cell differentiation and functional maturation. We found PPAR*β/δ* expression selectively upregulated in mouse embryonic pancreases or stem cells-derived INS^+^ cells at the pancreatic mature stage *in vivo* and *in vitro*. Strikingly, given the inefficiency of generating INS^+^ cells *in vitro*, PPAR*β/δ* activation displayed increasing mouse and human ES cell-derived INS^+^ cell numbers and insulin secretion. This phenomenon was closely associated with the forkhead box protein O1 (Foxo1) nuclear shuttling, which was dependent on PPAR*β/δ* downstream PI3K/Akt signaling transduction. The present study reveals the essential role of PPAR*β/δ* activation on p-Foxo1/Foxo1 status, and in turn, determining INS^+^ cell generation and insulin secretion *via* affecting pancreatic and duodenal homeobox-1 expression. The results demonstrate the underlying mechanism by which PPAR*β/δ* activation promotes functional INS^+^ cell differentiation. It also provides potential targets for anti-diabetes drug discovery and hopeful clinical applications in human cell therapy.

Differentiation of embryonic stem (ES) cells into insulin-positive (INS^+^) cells offers an innovative approach to screen anti-diabetes drugs, supply donor *β*-cell sources for cell therapy of diabetes and reveal underlying mechanisms for induced pluripotent stem cell researches.^1, 2, 3^ However, the spontaneous generation of INS^+^ cells from ES cells happens at a low rate, and most of these induced cells show limited glucose-stimulated insulin secretion (GSIS), which is limited in basic and clinical applications.^4, 5^ Consequently, seeking crucial targets and related signaling pathway in functional INS^+^ cell differentiation has become an important and urgent topic.

Peroxisome proliferator-activated receptors (PPARs) are nuclear receptors that participate in lipid metabolism, mitochondrial function and cell differentiation. PPARs may be involved in maintaining successful pregnancy, and also play essential roles in diabetes therapy *via* their effects on non-pancreas tissues.^6, 7, 8, 9, 10, 11^ Although PPAR functioning as the sensor in fatty acid oxidation^12^ and mitochondrial oxidative phosphorylation is required for stem cell differentiation,^13^ the link between PPARs and INS^+^ cell differentiation is still unclear. Three PPAR subtypes, PPAR*α*, PPAR*β*/*δ* and PPAR*γ*, have diverse expression profiles and biochemical characteristics in different tissues.^8, 9^ In mature pancreatic *β*-cells, PPAR*β*/*δ* is highly expressed, whereas the levels of PPAR*α* and PPAR*γ* are relatively lower.^14, 15^ Functionally, both PPAR*α* and PPAR*β*/*δ* display a protective effect against metabolic stress in *β*-cells;^15, 16^ PPAR*γ* is required to maintain glucose metabolism, because PPAR*γ* reduction leads to abnormal glucose metabolism in islets.^17^ To date, little is known about PPAR expression and activation in the differentiation process of ES cell into INS^+^ cells. Thus, we hypothesize that PPAR activation might be required for the differentiation of pluripotent stem cell into INS^+^ cells through affecting related signaling transduction.

Forkhead box protein O1 (Foxo1) is a negative regulator of pancreatic and duodenal homeobox-1 (Pdx-1) in adult *β*-cells.^18^ Deficiency of Foxo1 improves glucose tolerance and *β*-cell neogenesis in high-fat high-sucrose feeding mice.^19^ Foxo1 protects against stress-induced *β*-cell failure through the induction of two insulin transcription factors—neurogenic differentiation 1 (NeuroD1) and v-maf musculoaponeurotic fibrosarcoma oncogene family, protein A (avian) (Mafa).^20^ Ablation of Foxo1 in *β*-cells leads to impaired insulin secretion^19^ and *β*-cell dedifferentiation^21^ under metabolic stress. These reports indicate that Foxo1 possesses diverse functions in pancreas at physiological or pathological conditions. Considering Pdx-1 is required for *β*-cell generation and maturation at embryonic period,^22^ we further assume that Foxo1 probably participates in the differentiation of ES cells into functional INS^+^ cells. PPARs are associated with Akt signaling^23, 24, 25^ and also interact with Foxo1 in various tissues.^26, 27, 28, 29^ Oppositely, in the regulation of muscle oxidative metabolism, PPAR*β*δ*δ* induces Foxo1 transcription without the involvement of PI3K pathway.^29^ Exogenous Pdx-1 expression in ES cells improves pancreatic cell lineage differentiation.^30^ To date, the possible signaling transduction of PPARs/Foxo1/Pdx-1 pathway has not been defined. On the basis of these observations, therefore, clarifying the specific network will help us to understand how PPARs may affect INS^+^ cell differentiation.

Both PPAR*α* and PPAR*γ* enhance Pdx-1 expression, but the outcome seems different. For example, PPAR*α*δPdx-1 displays an protecting effect against GSIS insult in rat isolated pancreatic islets and rat insulinoma cells.^31, 32^ However, PPAR*γ* improves *Pdx-1* transcription accompanied by reducing insulinoma cell numbers without affecting Pdx-1 protein expression and GSIS function.^31, 32^ It implies that diverse regulating links may exist between different PPAR subtypes and Pdx-1. To date, it has not yet been revealed whether PPAR*β*/*δ* activation-induced Foxo1 shuttling associates with Pdx-1 in INS^+^ cell differentiation. PPAR*β*/*δ* modulates mitochondrial biogenesis and function,^7^ and Pdx-1 repression also results in mitochondrial dysfunction.^33^ We therefore explored the potential link of PPAR*β*/*δ*δFoxo1 and Pdx-1 in modulating INS^+^ cell differentiation.

Here, we demonstrate that PPAR*β*/*δ* activation is essential for modulating p-Foxo1/Foxo1 status, which contributes to the differentiation of ES cells into INS^+^ cells and insulin secretion. These results highlight the crucial aspects of PPAR*β*/*δ*/Foxo1 on the generation of functional INS^+^ cells. Therefore, the data may provide new insights into the underlying mechanisms by which PPAR*β*/*δ* modulates functional INS^+^ cell differentiation from induced pluripotent stem cells. These results may also help the development of anti-diabetes drugs.^34, 35^

## Results

### PPAR*β*/*δ* are highly expressed in mouse ES cell-derived INS^+^ cells

**Figure 1:**
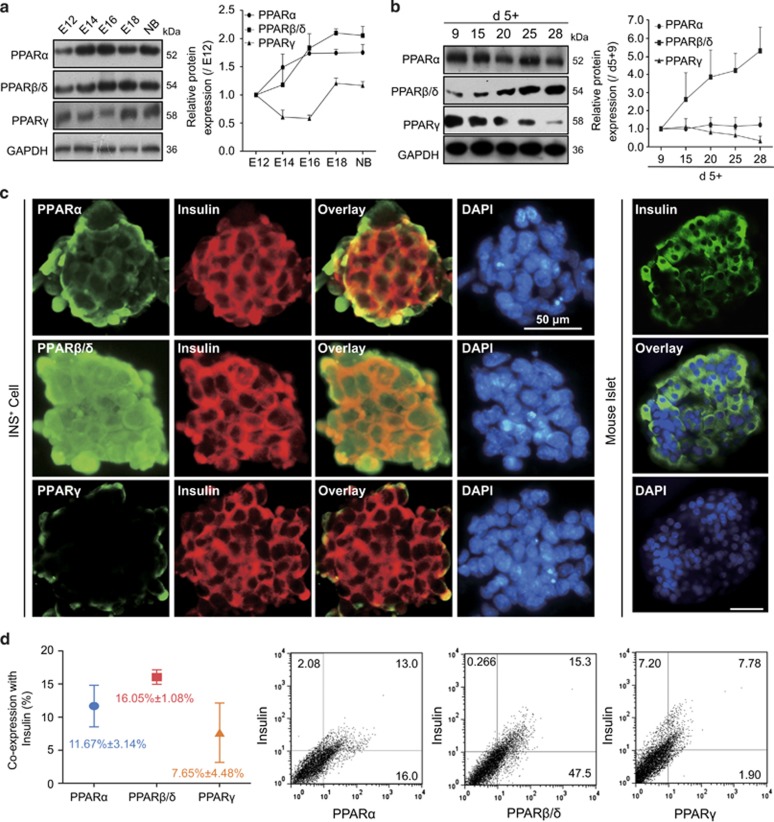
PPARs expressions at pancreatic mature stage *in vivo* and *in vitro*. (**a**) PPAR protein expressions in pancreas at embryonic days E12, 14, 16, 18 of gestation and newborn mouse, *n*=3. (**b**) PPARs expressed during INS^+^ cell differentiation stage, *n*=3. (**c**) Co-expressions of PPARs with insulin at terminal day (left panel), and insulin expression in mouse isolated islets (right panel) were determined by Immunofluorescence staining. Bar=50 *μ*m. (**d**) Flow cytometry assay demonstrated the PPARs were co-expressed with insulin at the terminal differentiation, *n*=4. Values represent mean ±S.D.

To evaluate the expression of PPARs in INS^+^ cell differentiation, we first compared their expressions in mouse embryonic pancreas *in vivo* (Figure 1a). PPAR*β*/*δ* displayed a robust increase from embryonic day E12 to E18 of gestation, and remained almost the same level to newborn pancreas. PPAR*α* only showed a slow upregulation. PPAR*γ* expression descended from E12 to E16 and then tuned to a higher expression level at E18. The results implied that PPARs might be important regulators in mouse embryonic *β*-cell development.

We further explore the presence of PPARs in ES cell-derived INS^+^ cell differentiation *in vitro*. Differentiated INS^+^ cells from mouse ES cells were harvested according to the three-stage protocol of Schroeder *et al.*^36^ The mRNA levels of the islet precursor cell marker *Neurogenin 3* (*Ngn3*), the *β*-cell differentiation markers *NeuroD1*, *Paired box 4* (*Pax4*) and *NK6 homeobox 1* (*Nkx6.1*), and the *β*-cell maturation markers *Glucose transporter 2* (*Glut-2*) *and Zinc transporter 8* (*ZnT8*) were confirmed at each differentiation stage. As shown in Supplementary Figure S1, *Ngn3* exhibited a peak expression at the initiation of the third stage; *NeuroD1*, *Pax4*, *Nkx6.1*, *Glut-2* and *ZnT8* expressions were gradually increased following the *Ngn3* expression (Supplementary Figure S1). Meanwhile, the insulin content of induced cells was glucose concentration-dependent (Supplementary Figure S2). All these data suggested that the mature INS^+^ cells were generated from mouse ES cells. Expressions of PPARs were detected at the third INS^+^ cell differentiation stage. Western blot indicated that PPAR*β*/*δ* expression was increased in a time-dependent manner. However, PPAR*α* expression was sustained at a relatively steady level, whereas PPAR*γ* expression showed a decrease in levels (Figure 1b).

Immunofluorescence imaging analysis showed that insulin expressed at the terminal day of differentiation, in a manner similar to that of mouse isolated islets (Figure 1c). Each PPAR subtype was expressed in induced cells, PPAR*β*δ*δ* was well co-expressed with insulin (Figure 1c). Flow cytometry assay confirmed the co-expression rates in parallel, the ratios of PPAR*α*, PPAR*β*/*δ* and PPAR*γ* with insulin were 11.67%, 16.05% and 7.65% at terminal differentiation, respectively (Figure 1d). These results suggested that PPAR*β*/*δ* may play a more important role than the other two members in INS^+^ cell differentiation.

### PPAR*β*/*δ*/Pdx-1 promoted functional INS^+^ cell differentiation

**Figure 2:**
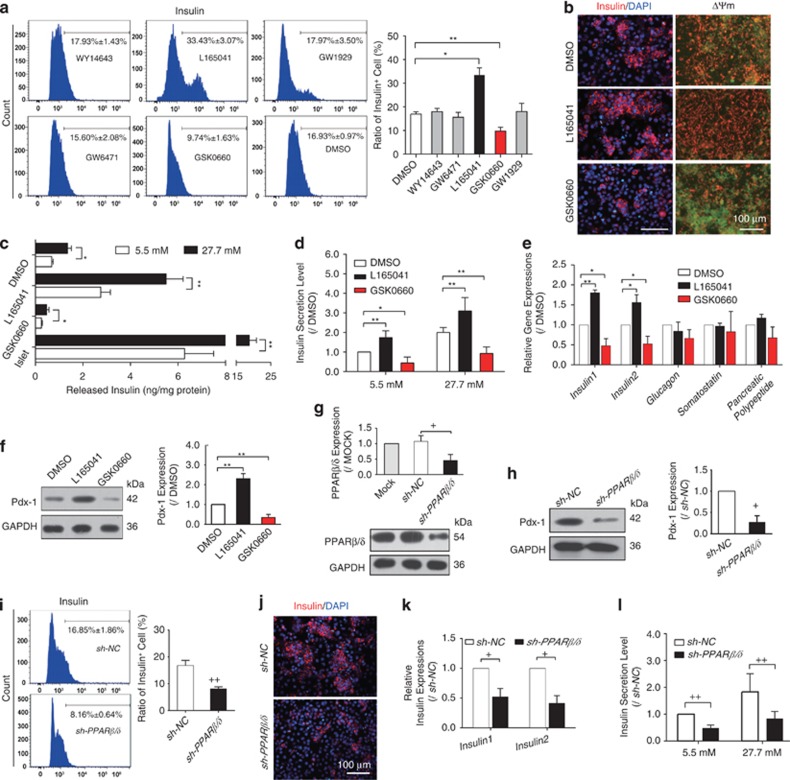
PPAR*β*/*δ* determined Pdx-1 expression and functional INS^+^ cell generation from mouse ES cells. Cells were treated with PPARs agonists, antagonists from day 5+9, or transfected with sh-PPAR*β/δ* or sh-NC at day 5+9 and collected at day 5+28 for further detection. PPAR*α* agonist: WY14643; PPAR*α* antagonist: GW6471; PPAR*β*δ*δ* agonist: L165041; PPAR*β*δ*δ* antagonist: GSK0660; PPAR*γ* agonist: GW1929. (**a**, **i**) Ratios of INS^+^ cells at day 5+28 were detected by flow cytometry analysis. (**a**, *n*=3, **i**, *n*=4.) (**b**) PPAR*β*δ*δ* changed the insulin-staining area and ΔΨm. Bar=100 *μ*m. (**c**) Released insulin of induced cells and isolated mouse islets were analyzed. Values were normalized with total protein contents, *n*=3. (**d,** **l**) Insulin secretion level of INS^+^ cells was measured at terminal day of differentiation. (**d**, *n*=5, **l**, *n*=3). (**e**, **k**) Pancreatic specific gene expressions were detected by quantitative RT-PCR. *n*=3. (**f,** **h**) Western blot analyzed Pdx-1 protein expression at day 5+28, *n*=3. (**g**) PPAR*β*δ*δ* protein expression was receded till day 5+28 after sh-PPAR*β/δ* transfection, *n*=3. (**j**) Insulin staining area was reduced after transfected with sh-PPAR*β/δ*. Bar=100 *μ*m. Values represent mean ±S.D. Statistical significance was set as *P<0.05, **P<0.01 *versus* DMSO control, ^*+*^*P*<0.05, ^*++*^*P*<0.01 *versus* sh-NC control

We next investigated whether the activation of PPARs could affect INS^+^ cell differentiation. Treatment with the PPAR*β*/*δ* agonist L165041 considerably increased the ratio of differentiated INS^+^ cells by twofold, raising the ratio from 16.93 to 33.43%. In contrast, treatment with the antagonist GSK0660 decreased the ratio to 9.74%. In contrast, neither PPAR*α* agonist/antagonist, nor PPAR*γ* agonist affected INS^+^ cell formation (Figure 2a). Immunofluorescence morphological analysis showed that PPAR*β*/*δ* activation increased the number of INS^+^ cells (Figure 2b). Considering that PPAR*β*/*δ* is correlated with mitochondrial function in ES cell differentiation,^37^ we detected the change of mitochondrial membrane potential (ΔΨm) in PPAR*β*/*δ*-mediated INS^+^ cell differentiation. As a result, PPAR*β*/*δ* activation was involved in maintaining mitochondrial ΔΨm. The red fluorescence was enhanced by L165041 but attenuated by GSK0660 (Figure 2b).

Meanwhile, we analyzed the GSIS function of induced cells. Insulin release stimulated by glucose was increased in PPAR*β*/*δ*-activated cells, which resembled that of isolated mouse islets (Figure 2c). Although GSK0660-treated cells were still glucose-responsive, they presented impaired insulin secretion function by releasing insulin at a lower efficiency (Figure 2c). Considering the discrepancy in INS^+^ cell populations among these groups, we also evaluated insulin secretion level of INS^+^ cells, which was defined as the ratio of released insulin to INS^+^ cell rates. As a result, insulin secretion level was increased by L165041 but declined by GSK0660 after glucose stimulation (Figure 2d). Meanwhile, PPAR*α* or PPAR*γ* activation did not affect the GSIS function of induced INS^+^ cells (Supplementary Figure S3a). In addition, *insulin-1* and *insulin-2* expressions were considerably elevated by L165041 but repressed by GSK0660. In contrast, the expressions of *α* cell marker *glucagon*, *δ* cell marker *somatostatin* and PP cell marker *pancreatic polypeptide* were not affected by PPAR*β*/*δ* activation (Figure 2e). These data suggested that PPAR*β*/*δ* activation may increase INS^+^ cell generation and insulin secretion accompanied by affecting the ΔΨm and *insulin* transcription.

We next examined the effect of PPAR*β*/*δ* activation on the expression of Pdx-1, the regulator of *β*-cell generation and function.^22, 33^ As shown in Figure 2f, PPAR*β*/*δ* agonist L165041 increased Pdx-1 expression, whereas the antagonist GSK0660 repressed its expression, indicating that PPAR*β*/*δ* may be the upstream event of Pdx-1. In contrast, neither PPAR*α* nor PPAR*γ* activation affected the expression level of Pdx-1 during the differentiation (Supplementary Figure S3b).

To further confirm the effects of PPAR*β*/*δ* in INS^+^ cell differentiation, PPAR*β*/*δ* was inhibited by shRNA at the indicating stage. PPAR*β*/*δ* expression dropped to 46% in shRNA interfered cells (Figure 2g). Knockdown of *PPARβ/δ* also decreased Pdx-1 expression (Figure 2h) followed by a decrease in INS^+^ cells ratio from 16.85 to 8.16% (Figure 2i), and a reduced insulin staining area (Figure 2j). Moreover, knockdown of *PPARβ/δ* reduced *insulin-1* and *insulin-2* transcriptions by 48 and 59% (Figure 2k), respectively, and impaired GSIS function of INS^+^ cells (Figure 2l). Taken together, these results suggest that PPAR*β*/*δ* may play an important role in promoting functional INS^+^ cell differentiation, which might be mediated by Pdx-1 expression.

### PPAR*β*/*δ* activation affected Foxo1 status, Gsk3*β* and their upstream signaling molecules

**Figure 3:**
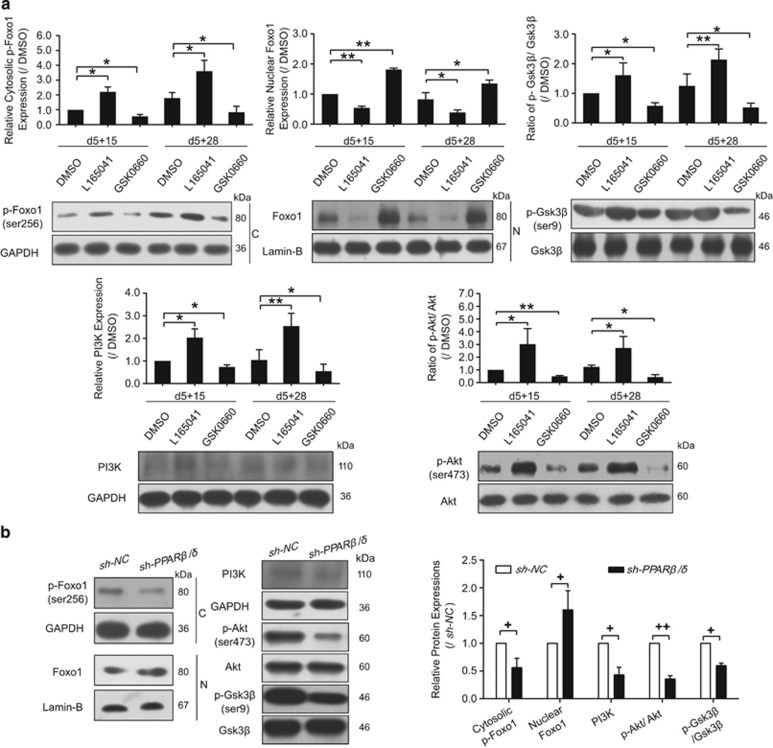
Foxo1, Gsk3*β* and PI3K/Akt signaling were associated with PPAR*β*δ*δ* activation. (**a**) Protein expressions at day 5+15 and day 5+28 were detected after L165041 or GKS0660 treatment from day 5+9. (**b**) Protein expressions at day 5+28 were analyzed after PPAR*β*δ*δ* shRNA transfection from day 5+9. Values represent mean ±S.D., *n* =3. Statistical significance was set as *P<0.05, **P<0.01 *versus* DMSO control, ^*+*^*P*<0.05, ^*++*^*P*<0.01 *versus* sh-NC

Given the observation that Pdx-1 may mediate the effect of PPAR*β*/*δ* in INS^+^ cell differentiation, we next analyzed the expressions and distribution of its negative regulator Foxo1 and Gsk3*β.*^38^ We also explored the network of PPAR*β*/*δ* with the upstream signaling of Foxo1 and Gsk3*β*, PI3K/Akt, during pancreatic differentiation stage. Phosphorylated Foxo1 (p-Foxo1) is a nucleocytoplasmic shuttling protein, and we found that the expression levels of cytosolic p-Foxo1, p-Gsk3*β*, PI3K and p-Akt were all affected by PPAR*β*/*δ* activation (Figure 3a). In contrast, the nuclear Foxo1 level displayed the opposite phenomenon after PPAR*β*/*δ* activation (Figure 3a). The knockdown of *PPARβ/δ* in the cells was consistent with the treatments of PPAR*β*/*δ* antagonist. Expressions of PI3K, p-Akt, p-Gsk3*β,* and cytosolic p-Foxo1 were all downregulated after transfection with sh-PPAR*β/δ*, whereas the nuclear Foxo1 level was increased (Figure 3b).

All these results indicate that PPAR*β*/*δ* activation could regulate the p-Foxo1/Foxo1 status, Gsk3*β* phosphorylation and PI3K/Akt signaling pathway, which might serve as a switch in controlling INS^+^ cell differentiation and insulin secretion.

### Knockdown of *Foxo1* improved PPAR*β*/*δ*-mediated cell differentiation

**Figure 4:**
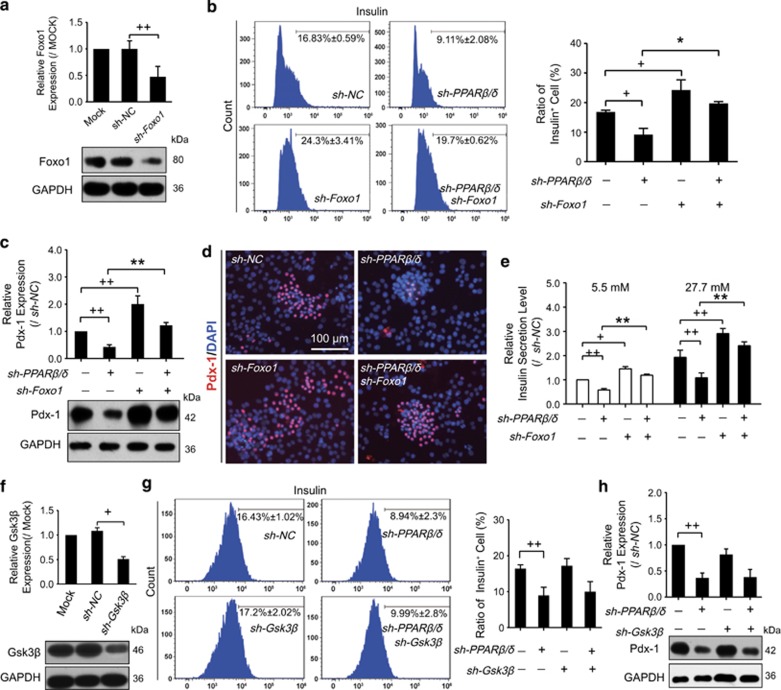
Knockdown of *Foxo1* improved PPAR*β*/*δ*-mediated cell differentiation. Cells were transfected with shRNA against PPAR*β*/*δ*, Foxo1 and Gsk3*β* from day 5+9 and harvested at day 5+28. (**a**, **f**) Protein expressions of Foxo1 (**a**) and Gsk3*β* (**f**) were repressed till 5+28 after transfection by targeted *shRNA*. (**b**, **g**) Ratios of INS^+^ cells after transfection were determined by flow cytometry. (**c**, **d** and **h**) Pdx-1 expression was determined by western blot (**c**, **h**) and immunofluorescence analysis (**d**, Bar= 100 *μ*m). (**e**) Insulin secretion levels of INS^+^ cells detected by ELISA. Values represent mean ±S.D., *n*=3. Statistical significance was set as ^+^*P*<0.05, ^++^*P*<0.01 *versus* sh-NC, *P<0.05, **P<0.01 *versus* sh-PPAR*β/δ*

To elucidate the mechanisms underlying PPAR*β*/*δ*-mediated INS^+^ cell differentiation, we further investigated the roles of Foxo1 and Gsk3*β* at the pancreatic differentiation stage. Foxo1 expression dropped by 52% in shRNA-interfered cells (Figure 4a). Flow cytometry analysis showed that sh-Foxo1 transfection increased the ratio of INS^+^ cells from 16.83 to 24.3% (Figure 4b). Meanwhile, in sh-PPAR*β/δ*-transfected cells, *Foxo1* knockdown reversed the inhibitory effect on INS^+^ cell generation from 9.14 to 19.7% (Figure 4b). In addition, sh-Foxo1 increased Pdx-1 expression. Even in the *PPARβ/δ* knockdown cells, the decreased Pdx-1 was upregulated by transfection of sh-Foxo1 (Figures 4c and d). ELISA demonstrated that sh-Foxo1 resulted in enhanced INS^+^ cell insulin secretion. Knockdown of *Foxo1* improved the sh-PPAR*β/δ* -induced low insulin levels after incubation with glucose (Figure 4e). The data suggest that Foxo1 regulates the insulin secretory ability of PPAR*β*/*δ*-induced INS^+^ cells.

In contrast, although Gsk3*β* expression was reduced to 51% in sh-Gsk3β-treated cells (Figure 4f), neither INS^+^ cell population nor Pdx-1 expression was altered after sh-Gsk3β transfection (Figures 4g and h). We therefore confirm that it might be Foxo1 rather than Gsk3*β* that participated in PPAR*β*/*δ*-mediated differentiation of INS^+^ cells.

Foxo1 modulated two *β*-cell development regulators NeuroD1 and Mafa;^20, 39^ therefore, we further explored whether PPAR*β*/*δ* activation was associated with the mRNA expression levels of these two factors during differentiation. As a result, neither *NeuroD1* nor *Mafa* was altered by PPAR*β*/*δ* activation or inhibition (Supplementary Figure S4). Thus, it implied that PPAR*β*/*δ* activation controlled p-Foxo1/Foxo1 status, which regulated INS^+^ cell differentiation through Pdx-1 signaling without the involvement of NeuroD1 or Mafa signaling.

### PI3K/Akt pathway is involved in PPAR*β*/*δ*/Foxo1δPdx-1-mediated cell differentiation

**Figure 5:**
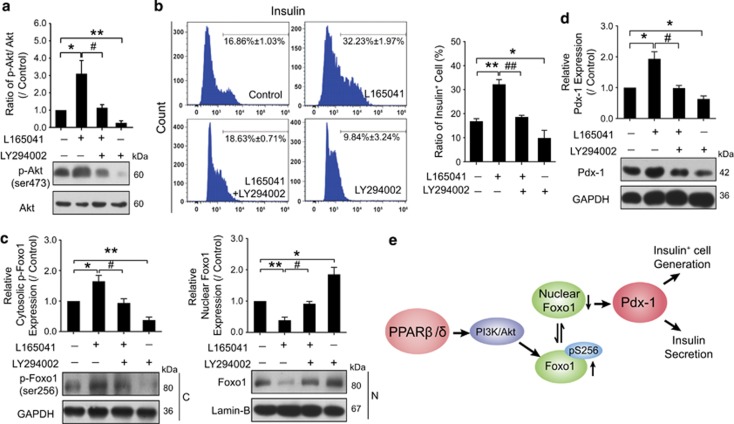
Inhibition of PI3K disturbed the effects of PPAR*β*δ*δ* on INS^+^ cell differentiation. Cells were treated with L165041 and LY294002 from day 5+9, and harvested at day 5+28 for further analysis. (**a**) LY294002 inhibited p-Akt expression. (**b**) The ratios of INS^+^ cells were determined by flow cytometry. (**c**) Expressions of cytosolic p-Foxo1 and nuclear Foxo1 were changed after treatment with LY294002. (**d**) Protein expression of Pdx-1 was analyzed by western blot. Values represent mean ±S.D., *n* =3. Statistical significance was set as *P<0.05, **P<0.01 *versus* control, ^#^*P*<0.05*,* ^##^*P*<0.01 *versus* L165041-treated groups. (**e**) Schematic diagram of the signaling pathways involved in PPAR*β*δ*δ*-mediated INS^+^ cell differentiation of mouse ES cells

To identify how PPAR*β*/*δ* regulated Foxo1 expression, we investigated the role of PI3K/Akt pathway in INS^+^ cell differentiation. Cells were treated with PI3K inhibitor LY294002 in the presence of PPAR*β*/*δ* agonist L165041 at the indicating stage. As a result, phosphorylation of Akt was remarkably decreased after PI3K inhibition by LY294002 (Figure 5a). Flow cytometry demonstrated that PI3K/Akt inhibition reduced the ratio of INS^+^ cells from 16.86 to 9.84% compared with DMSO control, and significantly blocked the promoting effect of PPAR*β*/*δ* activation by reducing the percentage of INS^+^ cells from 32.23 to 18.63% (Figure 5b). Importantly, the suppression of PI3K/Akt pathway decreased the cytosolic p-Foxo1 level accompanied by increased nuclear Foxo1 levels in both DMSO- and L165041-treated cells, indicating that PI3K/Akt pathway may be involved in the regulation of p-Foxo1/Foxo1 status by PPAR*β*/*δ* activation during INS^+^ cell differentiation (Figure 5c), in turn influencing Pdx-1 expression and function (Figure 5d). It implies that, unlike the regulation in muscle oxidative metabolism,^29^ p-Foxo1/Foxo1 status plays a role in the regulation of PPAR*β*/*δ*/Pdx-1 for INS^+^ cell generation *via* PI3K/Akt signaling transduction. In contrast, neither PPAR*α* nor PPAR*γ* activation influenced the p-Foxo1/Foxo1 status and PI3K/Akt pathway (Supplementary Figure S5), which therefore further confirmed that it was the PPAR*β*/*δ* isoform that has a role in promoting functional INS^+^ cell generation.

### Human ES cell-derived functional INS^+^ cells share the PPAR*β*/*δ* pathway during differentiation

**Figure 6:**
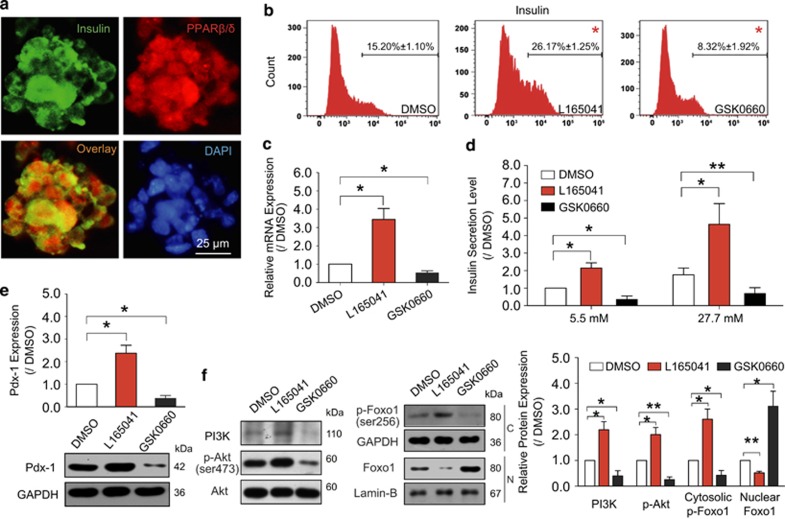
Human ES cell-derived INS^+^ cells share the same function and signaling pathway of PPAR*β*/*δ* activation. (**a**) Co-expressions of PPAR*β*/*δ* with insulin in human ES cell-derived INS^+^ cells at the terminal differentiation day. Bar=25 *μ*m. Human ES cells were treated with PPAR*β*/*δ* agonist L65041 or antagonist GSK0660 at INS^+^ cell differentiation stage, and the results shown on the terminal day are as follows. (**b**) The INS^+^ cells were quantified by flow cytometry assay. (**c**) Expression of *insulin* mRNA was detected by quantitative RT-PCR. (**d**) Insulin secretion level of INS^+^ cells was measured. (**e**, **f**) The molecular events in the PPAR*β*/*δ* signaling pathway demonstrated similar characteristics as those in mouse-ES cell-derived INS^+^ cells, *n*=3. Values represent mean±S.D. Statistical significance was set as *P<0.05, **P<0.01 *versus* DMSO control

To explore whether the PPAR*β*/*δ* pathway was involved in the differentiation process of human ES cell-derived INS^+^ cells, the human cell line H9 was employed and evaluation was performed according to a previous protocol.^40^ At the terminal differentiation day, immunofluorescence analysis showed that PPAR*β*/*δ* was well co-expressed with insulin in the cells (Figure 6a). Additionally, flow cytometry assay demonstrated that the co-expression ratio of PPAR*β*/*δ* and insulin was 14.4%, indicating that more than 93% INS^+^ cells expressed PPAR*β*/*δ* at the terminal differentiation (Supplementary Figure S6).

We then investigated whether PPAR*β*/*δ* activation could also promote human ES cells to differentiate into functional INS^+^ cells during the differentiation period. As a result, PPAR*β*/*δ* agonist L165041 increased the ratio of differentiated INS^+^ cells from 15.2 to 26.17% conversely, treatment with PPAR*β*δ*δ* antagonist GSK0660 decreased the ratio to 8.32% (Figure 6b). *Insulin* mRNA expression was also upregulated in PPAR*β*/*δ*-mediated human ES cell differentiation (Figure 6c). Meanwhile, insulin secretion level was increased by L165041 but reduced by GSK0660 after glucose stimulation (Figure 6d). In addition, Pdx-1 expression was modulated by PPAR*β*δ*δ* activation as well (Figure 6e). All these results implied that human ES cell-derived INS^+^ cells shared PPAR*β*/*δ* signaling pathway with mouse ES cells in differentiation. Moreover, expression levels of all the events in the pathway, PI3K, p-Akt and cytosolic p-Foxo1, were increased in PPAR*β*δ*δ*-mediated differentiation, while the nuclear Foxo1 was decreased after PPAR*β*δ*δ* activation (Figure 6f). As shown in Figure 6, p-Foxo1/Foxo1 status was also involved in the regulating effect of PPAR*β*/*δ* on human ES cell-derived INS^+^ cells.

## Discussion

The present study demonstrates that PPAR*β*/*δ* activation plays a crucial role in controlling the differentiation of ES cells into functional INS^+^ cells. The time-dependent increase in PPAR*β*/*δ* suggests that PPAR*β*/*δ* may be a major player in functional INS^+^ cell development during the third INS^+^ cell differentiation stage. During this period, the highly expressed islet precursor marker was decreased, *β*-cell differentiation and maturation markers were in turn expressed, indicating that the islet progenitor cells gradually develop into INS^+^ cells and are ready to secrete insulin. The increased expression tendency of PPAR*β*/*δ* is well matched with that in mouse embryonic pancreas at the late development stage in the present study. High expression of PPAR*β*/*δ* in embryonic pancreas and mouse ES cells-induced INS^+^ cells might be essential for INS^+^ cell differentiation *in vivo* and *in vitro*. Our results indicate that PPAR*β*/*δ* acts as a unique promoter for INS^+^ cell differentiation. In contrast, this novel phenomenon is opposite to what happens in mice with mature pancreas. Knockdown of *PPARβ/δ* in epithelial compartment of the mouse pancreas increased islet numbers and enhanced insulin secretion in the mutant mice after weaning.^41^ We consider that the differences between ES cells and cells in mature pancreas are partly due to the difference in the mitochondrial-dependent energy generation during developmental embryonic period. Increased mitochondrial oxidative phosphorylation is required for pluripotent stem cell differentiation.^12, 13^ PPARs regulate transcription of target genes related to mitochondrial biogenesis and oxidative phosphorylation at ES cell differentiation course.^13, 42^ Mitochondrial ΔΨm status reflects the oxidative phosphorylation function in stem cells. Our results demonstrate that PPAR*β*/*δ* activation maintains higher mitochondrial ΔΨm state in differentiated INS^+^ cell, thereby promoting the INS^+^ cell maturation at pancreatic differentiation stage.

Foxo1 negatively regulated Pdx-1, and a gain-of-function *Foxo1* mutation resulted in impaired *β*-cell compensation owing to decreased Pdx1 expression.^43^ Foxo1 displayed distinct effects on pancreas in diverse genetic conditions, and the discrepancy was due to the difference in basal Pdx-1 expression levels.^19^ In *db/db* mice, Pdx-1 expression was well maintained, Foxo1 ablation impaired insulin secretion.^19^ On the contrary, in IRS2 KO mice, Pdx-1 expression was reduced, Foxo1 haploinsufficiency reversed *β*-cell failure by increasing Pdx-1 expression.^44^ Moreover, Foxo1 is associated with *β*-cell dedifferentiation under physiologic stress, however, these effects are due to Foxo1 nuclear localization in *β*-cells under metabolic stress.^21^ While in basal condition, Foxo1 deletion does not impair *β*-cell morphology or function.^21^ Here, we demonstrated that PPAR*β*/*δ* regulated p-Foxo1/Foxo1 status during INS^+^ cell differentiation. PPAR*β*/*δ* activation increased cytosolic p-Foxo1, which resulted in the decrease of nuclear Foxo1, thereby leading to the inactivation of Foxo1. Furthermore, Foxo1 protected against *β*-cell failure through its upregulating effect on NeuroD1 and MafA, however, the effect occured only under the condition that Foxo1 translocated to nucleus in response to *β*-cell oxidative stress.^20^ Conversely, Foxo1 knockin mice with specific activation in both the hypothalamus and pancreas showed decreased *NeuroD1* and *Mafa* expression in islets.^39^ Here we found that neither *NeuroD1* nor *Mafa* was associated with PPAR*β*/*δ* activation-controlled p-Foxo1/Foxo1 status, suggesting that Foxo1 regulated INS^+^ cell differentiation without the involvement of NeuroD1 or Mafa. In the regulation of muscle oxidative metabolism, PPAR*β*/*δ* induces Foxo1 transcription without the involvement of PI3K pathway.^29^ However, in INS^+^ cell differentiation process, we have found that PPAR*β*/*δ*-activated PI3K/Akt pathway phosphorylated cytosolic Foxo1, thus disturbing the translocation of Foxo1 to nucleus. On the basis of these observations, we conclude that PPAR*β*/*δ* activation negatively modulates Foxo1 through PI3K/Akt signaling pathway during INS^+^ cell differentiation.

Pancreatic islet contains *α*, *β*, *δ* and PP cells. We found that only *β*-cell-specific genes *insulin1* and *insulin2* were exclusively modulated by PPAR*β*/*δ*. Pdx-1 is a pancreas-specific homeoprotein, specifically localizing to pancreatic progenitor cells and mature *β*-cells. It designates the pancreas location in early embryos, and acts as a definitive factor for proper differentiation and maturation of pancreatic *β*-cells by stimulating insulin gene transcription.^22^ Considering Pdx-1 is essential for functional *β*-cell generation,^22, 45, 46^ we hypothesize that Pdx-1 could be a key downstream regulator in PPAR*β*/*δ*-induced INS^+^ cell generation. The expression of Pdx-1 was increased after PPAR*β*/*δ* activation and decreased after PPAR*β*/*δ* suppression, which acted in accordance with the INS^+^ cell population and insulin secretion. The observation that Pdx-1 can be upregulated by PPAR*β*/*δ* activation has not been reported, and our results establish a novel signaling connection in the PPAR*β*/*δ-*induced INS^+^ cell differentiation. *Foxo1* knockdown reversed the inhibitory effects on INS^+^ cell generation and insulin secretion caused by *PPARβ/δ* deficiency through the improvement of Pdx-1 expression, indicating that PPAR*β*/*δ* regulated Pdx-1 expression in functional INS^+^ cell differentiation *via* Foxo1 suppression. In addition, Gsk3*β* is another Pdx-1 negative regulator and inhibition of Gsk3*β* was reported to promote *β*-cell growth.^38^ However, in the present study, we did not find any effects on Pdx-1 expression or INS^+^ cell differentiation when Gsk3*β* is inhibited. Furthermore, neither PPAR*α* nor PPAR*γ* agonist affected the functional INS^+^ cell generation or the PI3K/Akt/Foxo1/Pdx-1 pathway activation, although PPAR*α* showed a considerable level during differentiation. In conclusion, Foxo1 is a major signaling molecule involved in PPAR*β*/*δ*/Pdx-1-promoted functional INS^+^ cell generation.

Most importantly, we further revealed that PPAR*β*/*δ* activation also exhibited its promoting effect on human ES cell-derived INS^+^ cell differentiation and insulin secretion via the same signaling pathway in mouse ES cell differentiation. Thus, PPAR*β*/*δ* expression or activation can serve as a pathological event in the mechanism evaluation of diabetes.

In summary, our study demonstrates that PPAR*β*/*δ* plays a crucial role in promoting ES cell-derived INS^+^ cell differentiation and insulin secretory capacity *via* affecting p-Foxo1/Foxo1 status (Figure 5e). The new finding sheds light on potential molecular signaling that influences INS^+^ cell differentiation in pluripotent stem cell research, pathological evaluation, and suggests a potential target for anti-diabetic drug development and hopeful clinical applications in human cell therapy.

## Materials and Methods

### INS^+^ cell differentiation of mouse and human ES cells

A three-step protocol^36^ was applied to induce INS^+^ cells from mouse ES-D3 cells (CRL-1934, American Type Culture Collection, Manassas, VA, USA). Embryoid bodies were aggregated by ES cells for 5 days, spontaneously generated three germ layers for 9 days in differentiation medium I and then differentiated into pancreatic lineage for another 19 days in differentiation medium II. The differentiation medium I consists of Iscove's modification of DMEM (IMDM, Life Technologies, Carlsbad, CA, USA), 20% FBS (Life Technologies), Glutamax (Life Technologies), non-essential amino acids (Life Technologies) and 450 *μ*M monothioglycerol (Sigma Aldrich, St. Louis, MO, USA), and differentiation medium II consists of DMEM/F12 (Life Technologies), 10 mM nicotinamide (Sigma Aldrich), 1 *μ*g/ml laminin (Sigma Aldrich), N2 media supplement (Life Technologies) and B27 media supplement (Life Technologies).

Human ES cell line H9 (from WiCell Research Insititute. Simple Letter Agreement: #10-W0353 to Yijia Lou) was differentiated into INS^+^ cells according to the protocol of Jiang *et al.*^40^ Human ES cells were plated into 1% Matrigel (BD Biosciences, San Jose, CA, USA)-coated dishes and cultured with chemically defined medium for 2 days. The chemically defined medium consists of 50% IMDM, 50% DMEM/F12, Insulin-Transferrin-Selenium-A (Life Technologies), 450 *μ*M monothioglycerol and 5 mg/ml albumin fraction V (Sigma Aldrich). Then, cells were induced to generate definitive endoderm with chemically defined medium containing 50 ng/ml activin A (Life Technologies) for 4 days, and cultured with chemically defined medium containing 10^−6^ M RA (Sigma Aldrich) to generate pancreatic progenitors for another 4 days. Progenitors were then cultured to achieve mature islets with islet maturation medium: DMEM/F12, Insulin-Transferrin-Selenium-A and 2 mg/ml albumin fraction V with 10 ng/ml bFGF (Life Technologies) for 3 days, with 10 mM nicotinamide for the next 5 days, and transferred into Ultra Low Attachment culture dishes for another 5 days in suspension culture after digested by Accutase (Millipore, Billerica, MA, USA).

### Chemicals treatments of cultures

Cells were treated with PPAR*α* agonist WY14643 (10 *μ*M, Sigma Aldrich), PPAR*α* antagonist GW6471 (1 *μ*M, Sigma Aldrich), PPAR*β*δ*δ* agonist L165041 (10 *μ*M, Sigma Aldrich), PPAR*β*δ*δ* antagonist GSK0660 (1 *μ*M, Sigma Aldrich), PPAR*γ* agonist GW1929 (10 *μ*M, Sigma Aldrich), PI3K inhibitor LY294002 (7.5 *μ*M, Cell Signaling Technology, Danvers, MA, USA) from day 5+9 to day 5+28 during mouse ES cell differentiation. For human ES cells, PPAR*β*δ*δ* agonist L165041 or antagonist GSK0660 were treated with the same concentration in mouse ES cells from differentiation day 10 to day 23. All these chemicals were dissolved in DMSO (Sigma Aldrich). Control condition was treated with vehicle DMSO (final concentration 0.1%).

### Fetal mouse pancreases obtain

ICR mice were obtained from the Experimental Animal Center, Zhejiang University, Hangzhou, Zhejiang, China (GradeI, Certificate No. 2007-0029). Ten-week-old ICR mice (4 female and 1 male) were housed together under a 12 h light/dark cycle. The day that vaginal sperm or a copulation plug was observed was defined as embryonic day 0 (E0) of gestation. Mouse embryonic pancreases were obtained at E12, E14, E16, E18 and from newborns.

### Flow cytometry analysis

Mouse ES or human ES differentiated cells at terminal differentiation day were digested into single cells with Accutase. After being fixed in 4% paraformaldehyde for 1 h at 4 °C, cells were blocked with 3% BSA for another 1 h at room temperature. Then, the cells were incubated at 4 °C overnight with primary antibodies: anti-insulin (1 : 200, Cell Signaling Technology), anti-PPAR*α* (1 : 200, Abcam, Cambridge, MA, USA), anti-PPAR*β*δ*δ* (1 : 200, Abcam) or anti-PPAR*γ* (1 : 200, Abcam). After that, cells were incubated with the appropriate secondary antibodies (1 : 500) for 20 min at 4 °C. Cells were collected with a FACS flow cytometer (Beckman Coulter, Carlsbad, CA, USA). The results were expressed as the percentage of the fluorescence intensity.

### Immunocytochemistry analysis

Isolated islets were obtained from 8–12-week-old male Balb/c mice (purchased from Experimental Animal Center, Zhejiang University, China, GradeI, Certificate No. 2008-0016) according to the protocol.^47^ After digesting from pancreases, islets were cultured on cover slips. Both ES cell lines on their terminal differentiation day and isolated islets were fixed with cold methanol for 10 min at −20 °C. Fixed cells were blocked with 10% FBS for 1 h at room temperature. After that, cells were incubated at 4 °C overnight with primary antibodies: anti-insulin (1 : 100), anti-PPAR*α* (1 : 100), anti-PPAR*β*δ*δ* (1 : 100), anti-PPAR*γ* (1 : 100) or anti-Pdx-1 (1 : 100, Cell Signaling Technology). Cultures were treated with appropriate secondary antibodies (1 : 400) for 2 h and DAPI (2 *μ*g/ml, Sigma Aldrich) for 1 min at room temperature. Finally, differentiated cells were observed under Leica DMI3000B microscope (Leica, Mannheim, Germany), and isolated islets were observed under Olympus FV1000 confocal microscope (Olympus, Tokyo, Japan). The overlay images were merged by software Image-Pro Plus.

### Western blot analysis

Total protein, cytosolic protein (exclusively for p-Foxo1) and nuclear protein (exclusively for Foxo1) from cells or tissues were harvested. Total proteins were obtained from samples by cell lysis buffer for western (Beyotime, Shanghai, China). Nuclear and cytoplasmic proteins were separated using Nuclear and Cytoplasmic Protein Extraction Kit (Beyotime) according to the manufacturer's instructions. Cells were dissolved with cytoplasmic protein extraction agent A and were vortexed for 10 s. Then, the cytoplasmic protein extraction agent B was added into the samples. After 5 s vortex and 5 s incubation on ice, the cells were centrifuged for 5 min at 12 000 × *g* at 4 °C, and the supernatant containing the cytosolic fraction was collected. The pellet was resuspended with nuclear protein extraction agent. After 15–20 times of vortexing for 30 min, the cells were centrifuged for 10 min at 12 000 × *g* at 4 °C, and the supernatant containing the nuclear extracts was collected. An aliquot of 20 *μ*g protein was loaded and separated on a SDS-polyacrylamide gel. After separation, proteins were transferred onto PVDF membranes. Then, the transferred membranes were blocked in 5% non-fat milk for 1 h and incubated at 4 °C overnight with primary antibodies: anti-PPAR*α* (1 : 1000), anti-PPAR*β*δ*δ* (1 : 1000), anti-PPAR*γ* (1 : 500), anti-Pdx-1 (1 : 1000), anti-Gsk3*β* (1 : 4000, Cell Signaling Technology), anti-p-Gsk3*β* (1 : 4000, Cell Signaling Technology), anti-p-Foxo1 (1 : 1000, Cell Signaling Technology), anti-Foxo1 (1 : 1000, Cell Signaling Technology), anti-GAPDH (1 : 5000, Santa Cruz Biotechnology, Santa Cruz, CA, USA), anti-PI3K (1 : 500, Santa Cruz Biotechnology), anti-lamin-B (1 : 500, Santa Cruz Biotechnology), anti-p-Akt (1 : 500, Santa Cruz Biotechnology), anti-Akt (1 : 1000, Santa Cruz Biotechnology). After three washes, the blots were incubated with secondary antibody (1 : 5000) for 1 h at room temperature. The proteins were visualized with an ECL (Pierce, Rockford, IL, USA). The density of the products was quantitated using image J software.

### Quantitative real-time RT-PCR

Total RNA was isolated from cells by Trizol reagent (Invitrogen, Carlsbad, CA, USA). Then, 1 *μ*g of RNA was treated by RT reagent kit (TAKARA, Dalian, China) according to the manufacturer's instructions. Amplifications were performed using SYBR premix ex taq kit (TAKARA). The sense and antisense primers were as shown in Table 1. Each measurement was normalized to *Gapdh* for each sample. The relative gene expression was presented by comparative C_T_ method.^48^

### Insulin secretion determination

Differentiated cells at terminal day were cultured without insulin for 3 h and thoroughly washed prior to ELISA. Differentiated cells or Groups of 20 similar sized islets were pre-incubated in KRBH buffer for 1 h at 37 °C. The medium was replaced with KRBH buffer containing either 27.7 mM glucose or 5.5 mM glucose for 1 h, and then the supernatant and cells for the determination of insulin secretory ability were collected. Media samples were analyzed using Rat/Mouse insulin ELISA kit (Millipore) or human insulin ELISA kit (Millipore). Released insulin was normalized to total protein content. The insulin secretion level was presented as the ratio of insulin secretion value to INS^+^ cell rate. Cells were detected by flow cytometry to evaluate the ratio of INS^+^ cells.

### Transfections with short hairpin RNAs (shRNA)

The shRNAs targeting mouse PPAR*β*δ*δ*, Gsk3*β*, Foxo1 mRNA and a negative control shRNA were purchased from Genpharma Corp (Shanghai, China) and are as follows: *PPARβ*δ*δ*: GGAGCATCCTCACCGGCAA and GCAGCTGGTCACTGAGCAT (1 : 1); *Gsk3β*: CATGAAAGTTAGCAGAGATAA; *Foxo1*: CGCCCCAGGTGGTGGAGAC; NC (Negative Control): GTTCTCCGAACGTGTCACGT. Digested cells at day 5+9 were transfected with either specific receptor shRNA or negative control shRNA at a final concentration of 1.6 *μ*g/ml with Lipofectamine 2000 transfection agent (Life Technology) for 24 h according to the manufacturer's protocol. To confirm the long-term silencing effect, protein levels of PPAR*β*δ*δ*, Gsk3*β* and Foxo1 were determined by western blot at day 5+28.

### Mitochondrial membrane potential (ΔΨm) assay

For the determination of ΔΨm, cells were incubated with 2 *μ*g/ml JC-1 (5,5′,6,6′-tetrachloro-1,1′,3,3′-tetraethyl-benzimidazolylcarbocyanine iodide, Sigma Aldrich) for 30 min at 37 °C in the dark. Cells were then washed by PBS and observed under Leica DMI3000B microscope. The red fluorescent J-aggregate indicates normal ΔΨm, while the green monomer fluorescence demonstrates low ΔΨm.

### Statistical analysis

Data are expressed as mean values±standard deviation (S.D.). At least three independent experiments were carried out as repeats. Statistical analysis was performed by student *t*-test when two groups were compared. When multiple groups were compared, ANOVA were used (GraphPad Prism 6; GraphPad Software Inc., San Diego, CA, USA). A value of *P*<0.05 was considered to be significant.

## Figures and Tables

**Table 1:** Primers and conditions for real-time RT-PCR

**Genes**	**Primers**	**Annealing temperature (°C)**
*Insulin 1 (Mouse)*	5′-CCAGCTATAATCAGAGACCA-3′ 5′-GTGTAGAAGAAGCCACGCT-3′	58
*Insulin 2 (Mouse)*	5′-CCCTGCTGGCCCTGCTCTT-3′ 5′-AGGTCTGAAGGTCACCTGCT-3′	58
*Glucagon*	5′-AAGGCGAGACTTCCCAGAAGA-3′ 5′-GCACGAGATGTTGTGAAGATGG-3′	58
*Pancreatic polypeptide*	5′-CTCCCTGTTTCTCGTATCCA-3′ 5′-TGTTCTCCTCCTCGGCTC-3′	55
*Somatostatin*	5′-TCGCTGCTGCCTGAGGACCT-3′ 5′-GCCAAGAAGTACTTGGCCAGTTC-3′	55
*Gapdh (Mouse)*	5′-TCCATGACAACTTTGGCATTG-3′ 5′- CAGTCTTCTGGGTGGCAGTGA-3′	58
*Insulin (Human)*	5′-GCAGCCTTTGTGAACCAACAC-3′ 5′-CCCCGCACACTAGGTAGAGA-3′	58
*Gapdh (Human)*	5′-CGGAGTCAACGGATTTGGTCGTAT-3′ 5′-AGCCTTCTCCATGGTGGTGAAGAC-3′	58

## Abbreviations

ES: embryonic stem
INS^+^: insulin-positive
GSIS: glucose-stimulated insulin secretion
PPARs: peroxisome proliferator-activated receptors
Foxo1: forkhead box protein o1
Pdx-1: pancreatic and duodenal homeobox-1
PI3K: Phosphoinositide 3-kinase
ΔΨm: mitochondrial membrane potential
shRNA: small hairpin RNA
NC: negative control
Gsk3*β*: glycogen synthase kinase 3*β*
Ngn3: Neurogenin 3
NeuroD1: Neurogenic differentiation 1
Pax4: Paired box 4
Nkx6.1: NK6 homeobox 1
Glut-2: Glucose transporter 2
ZnT8: Zinc transporter 8
protein A (avian) Mafa: v-maf musculoaponeurotic fibrosarcoma oncogene family
