# Supplementary material for: Peroxisome proliferator-activated receptorβ/δ activation is essential for modulating p-Foxo1/Foxo1 status in functional insulin-positive cell differentiation
Source: Cell Death Dis. 2015 Apr 9;6(4):e1715–. doi: 10.1038/cddis.2015.88 (PMC4650555; doi:10.1038/cddis.2015.88)
Supplement: Supplementary Information [file cddis201588x1.doc]

**Peroxisome proliferator-activated receptor β/δ activation is essential for modulating p-Foxo1/Foxo1 status in functional insulin-positive cell differentiation**

Lu Li^1^, Tong Li^1^, Yuyu Zhang^1^, Zongfu Pan^1^, Bowen Wu^1^, Xin Huang^2^, Yihan Zhang^1^, Yuqin Mei^1^, Lijun Ge^1^, Guofang Shen^1^, Ren-shan Ge^3,4^, Danyan Zhu^1^ and Yijia Lou^1^

^1^Institute of Pharmacology, Toxicology and Biochemical Pharmaceutics, Key Innovation Team for Stem Cell Translational Medicine of Cardiovascular Disease of Zhejiang Province, College of Pharmaceutical Sciences, Zhejiang University, Hangzhou, China. ^2^Cardiovascular Key Laboratory of Zhejiang Province, The 2nd Affiliated Hospital, College of Medicine, Zhejiang University, Hangzhou, China. ^3^The Population Council at the Rockefeller University, New York, NY 10021, USA. ^4^Institute of Reproductive Biomedicine, the 2nd Affiliated Hospital, Wenzhou Medical University, Wenzhou, China.

**Supplementary information**

This file includes:

Supplementary Methods

Supplementary Figures 1-6 and legends

Supplementary Table 1

**Supplementary Methods**

**Real-time RT-PCR.** Total RNA was isolated from cells by Trizol reagent (Invitrogen). Then 1μg of RNA was treated by RT reagent kit (TAKARA) according to the manufacturer’s instructions. Amplifications were performed using SYBR premix ex taq kit (TAKARA). The sense and antisense primers were as follows (Supplementary Table 1). Each measurement was normalized to *Gapdh* for each sample. The relative gene expression was presented by comparative C_T_ method.

**Insulin content and secretion determination.** Differentiated cells were pre-incubate in KRBH buffer for 1 h at 37°C. Replace the medium with KRBH buffer containing either 27.7 mM glucose or 5.5 mM glucose for 1 h, and then collect the supernatant and cells. Cells were treated with lysis buffer (Beyotime) for determination of insulin content and total intracellular protein. The insulin secretion value in sample medium and the insulin content in cells were determined by Rat/Mouse insulin ELISA kit (Millipore). Insulin content was normalized to total protein content. The insulin secretion level was presented as the ratio of insulin secretion value to INS^+^ cell rate. Cells were detected by Flow cytometry to evaluate the ratio of INS^+^ cells.

**Western Blot.** Total protein, cytosolic protein (exclusively for p-Foxo1) and nuclear protein (exclusively for Foxo1) from cells or tissues were harvested. Total proteins were obtained from samples by cell lysis buffer for western (Beyotime). The extraction and isolation of nuclear and cytosolic protein were by Nuclear and Cytoplasmic Protein Extraction Kit (Beyotime) according to the manufacturer’s instructions. An aliquot of 20 μg protein was loaded and separated on a SDS-polyacrylamide gel. After separation, proteins were transferred onto PVDF membranes. Then the transferred membranes were blocked in 5% non-fat milk for 1 h and incubated at 4°C overnight with primary antibodies: anti-Pdx-1 (1:1000), anti-p-Foxo1 (1:1000,Cell signaling technology), anti-Foxo1 (1:1000, Cell signaling technology), anti-GAPDH (1:5000, Santa Cruz Biotechnology), anti-PI3K (1:500, Santa Cruz Biotechnology), anti-lamin-B (1:500, Santa Cruz Biotechnology), anti-p-Akt (1:500, Santa Cruz Biotechnology), anti-Akt (1:1000, Santa Cruz Biotechnology). After three times wash, the blots were incubated with secondary antibody (1:5000) for 1 h at room temperature. The proteins were visualized with an ECL (Pierce). The density of the products was quantitated using software image J.

**Flow cytometry analysis.** human ES cell-differentiated cells at terminal differentiation day were digested into single cells with Accutase. After being fixed in 4% paraformaldehyde for 1 h at 4°C, cells were blocked with 3% BSA for another 1 h at room temperature. Then, the cells were incubated at 4°C overnight with primary antibodies: anti-Insulin (1:200, Cell signaling technology) and anti-PPARβ/δ (1:200, Abcam). After that, cells were incubated with the appropriate secondary antibodies (1:500) for 20 min at 4°C. Cells were collected with a FACS ﬂow cytometer (Beckman Coulter). The results were expressed as the percentage of the ﬂuorescence intensity.

**Supplementary Figures**


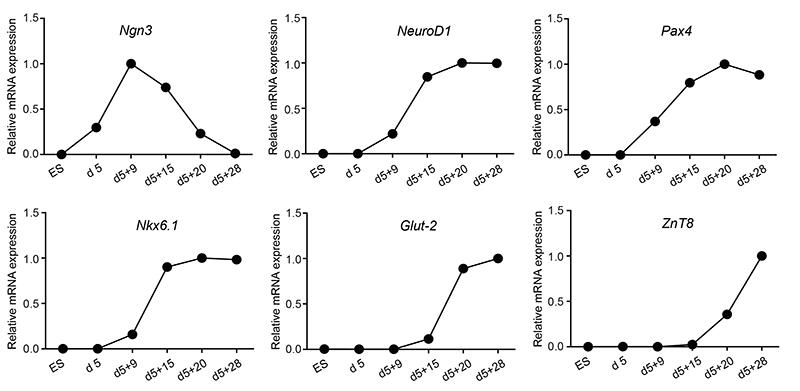


Supplementary Figure 1. The dynamic *β*-cell differentiation and maturation gene expression patterns during the differentiation of mouse ES cells into INS^+^ cells.


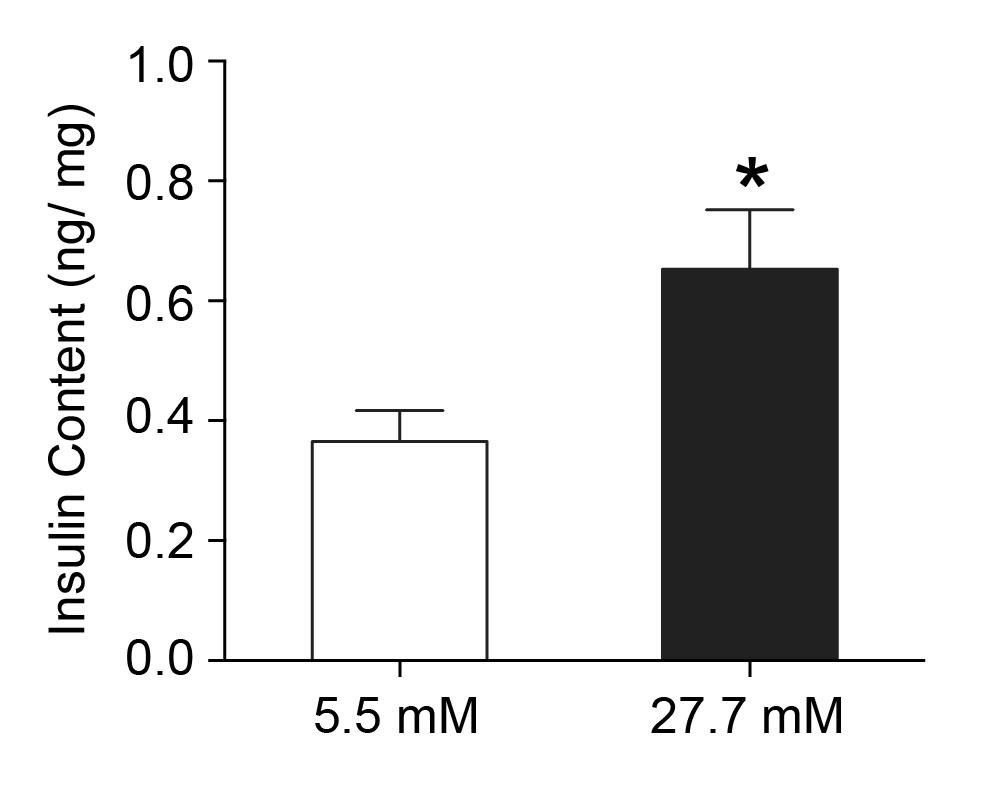


Supplementary Figure 2. The intracellular insulin content of differentiated mouse ES cells was responsive to glucose concentration. Values represent mean ±S.D. Statistical significance was set as **P*<0.05 versus 5.5 mM glucose concentration. *n*=3.


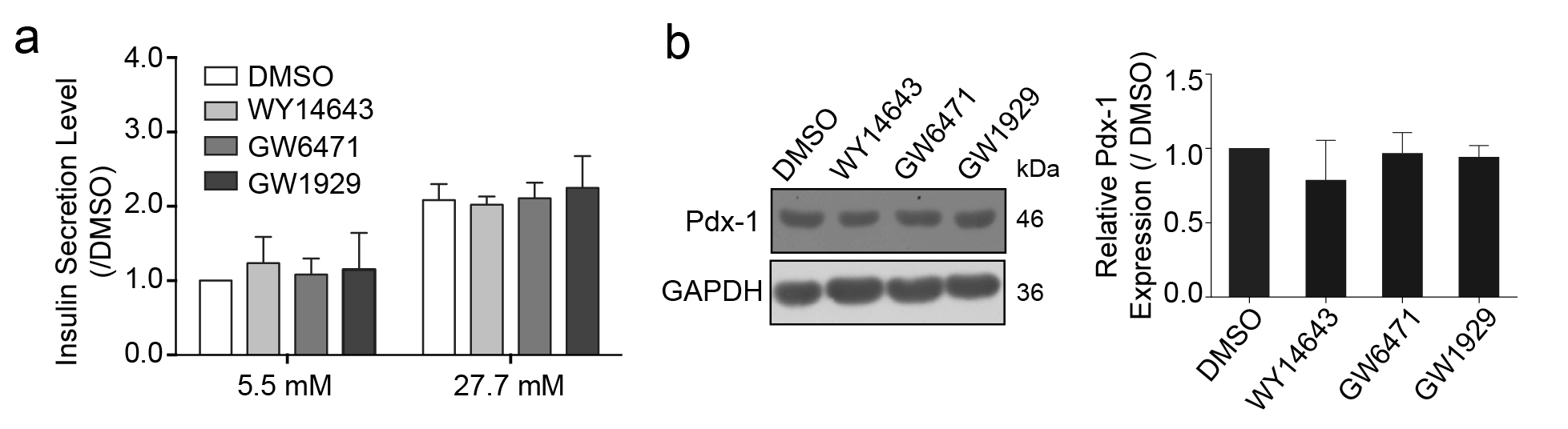


Supplementary Figure 3. PPARα agonist WY14643, antagonist GW6471 or PPARγ agonist GW1929 did not affect insulin secretion level (a) or Pdx-1 expression (b) during mouse ES cell-derived INS^+^ cell differentiation. *n*=3.


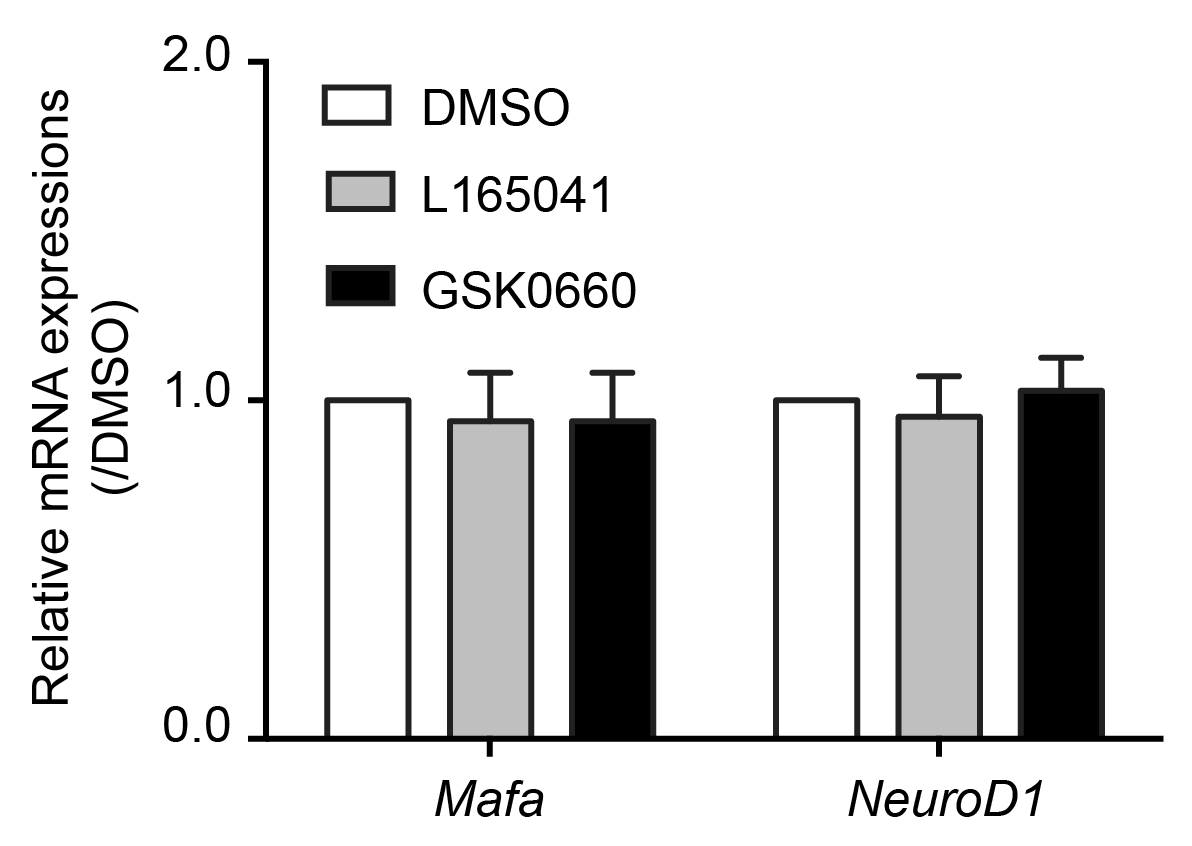


Supplementary Figure 4. PPARβ/δ agonist L165041 or antagonist GSK0660 did not affect mRNA expression levels of *Mafa* or *NeuroD1*. *n*=3.


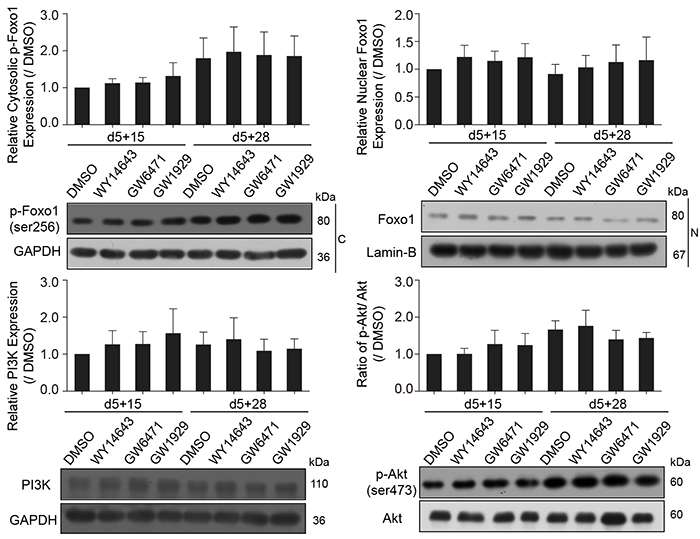


Supplementary Figure 5. PPARα agonist WY14643, antagonist GW6471 or PPARγ agonist GW1929 did not involve the PI3K/Akt/Foxo1 signaling pathway during mouse ES cell-derived INS^+^ cell differentiation. *n*=3.


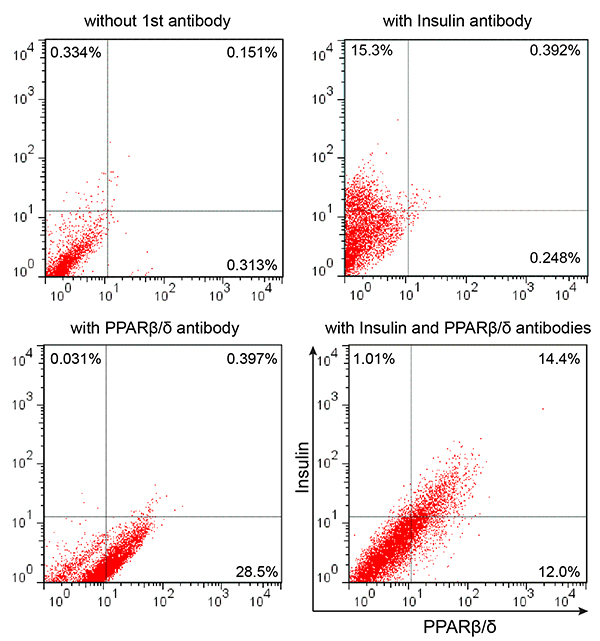


Supplementary Figure 6. Flow cytometry assay demonstrated that PPARβ/δ was co-expressed with insulin in the terminal differentiated human ES cells. The lower right panel exhibited the cell population of co-staining of PPARβ/δ and insulin, and the co-expression rate reached to 14.4%.

**Supplementary Table 1**

**Primers and conditions for Real-time RT-PCR**

| **Genes** | **Primers** | **Annealing temperature (°C)** |
| --- | --- | --- |
| *Ngn3* | 5’- CTTCGGAGAATGAGGAGGATGA-3’  5’- CCACCGCTCGATTTGTGCT-3’ | 58 |
| *NeuroD1* | 5’- CTTGGCCAAGAACTACATCTGG-3’  5’- GGAGTAGGGATGCACCGGGAA-3’ | 58 |
| *Pax4* | 5’- CTCCATCCAGAACCAGTCCCA-3’  5’- CCCCTAGCTGATTCACACTGC-3’ | 58 |
| *Nkx6.1* | 5’- CTTCGGAGAATGAGGAGGATGA-3’  5’- CCACCGCTCGATTTGTGCT-3’ | 58 |
| *Glut-2* | 5’- CGGTGGGACTTGTGCTGCTGG-3’  5’- CTCTGAAGACGCCAGGAATTCCAT-3’ | 58 |
| *ZnT8* | 5’- GTTGCTGGGAGTCTGGCTAT-3’  5’- TACCTGCTTGGATCTGGTAATC-3’ | 58 |
| *Mafa* | 5’-AGGAGGAGGTCATCCGACTGAA-3’  5’- CCCGCCAACTTCTCGTATTTCT-3’ | 58 |
| *Gapdh* | 5’- TCCATGACAACTTTGGCATTG-3’  5’- CAGTCTTCTGGGTGGCAGTGA-3’ | 58 |
